# Cytolethal distending toxin-producing *Escherichia coli* strains causing severe diarrhoea in young Mexican children

**DOI:** 10.1099/jmmcr.0.005079

**Published:** 2017-02-28

**Authors:** Mario Meza-Segura, Mussaret Bano Zaidi, Samantha Maldonado-Puga, Jazmin Huerta-Cantillo, Lucia Chavez-Dueñas, Fernando Navarro-Garcia, Teresa Estrada-Garcia

**Affiliations:** ^1^​ Department of Molecular Biomedicine, CINVESTAV-IPN, Mexico City, Mexico; ^2^​ Infectious Diseases Research Unit, Hospital General O’Horan, Merida, Mexico; ^3^​ Department of Epidemiology and Biostatistics, Michigan State University, Lansing, MI, USA; ^4^​ Department of Cell Biology, CINVESTAV-IPN, Mexico City, Mexico

**Keywords:** CDT-Positive *Escherichia coli*, severe diarrhoea, children

## Abstract

**Introduction.** Cytolethal distending toxins (CDTs), encoded by *cdt* genes, have DNase activity leading to cellular and nuclear distension, resulting in irreversible cell cycle arrest and apoptosis of target cells. *cdt*-positive *Escherichia coli* strains have been isolated from children with diarrhoea. There is, however, scant information on the prevalence and clinical presentation of diarrhoeal disease caused by these strains. Furthermore, toxin production of *cdt*-positive strains is rarely confirmed. We report five young children with diarrhoea caused by CDT-producing *E. coli* in whom stools were negative for other bacterial or enteric pathogens.

**Case presentation.** On admission to hospital, all children presented watery diarrhoea with high stool output (range 7–20 stools/24 h); five had fever of 38 °C or more and four presented vomiting. Dehydration was present in four patients, one of whom had hypovolaemic shock; one child also presented hyponatraemia and hypokalaemia. In two children, *cdt*-positive strains were classified as typical and atypical enteropathogenic *E. coli*, and the remaining three harboured *cdt*-positive strains that did not belong to any diarrhoeagenic pathogroup. One *cdt*-positive strain from each case was characterized by a CDT cytotoxic assay and a *cdt* type-specific PCR. All strains produced the characteristic cellular intoxication due to CDT. Two strains carried the *cdt*-*I*, one *cdt*-*III*, one *cdt*-*IV*, and one concurrently had *cdt-I, cdt-II* and *cdt*-*III* genes.

**Conclusion.** Our results suggest that CDT-producing *E. coli* strains are an infrequent, albeit significant, cause of severe diarrhoeal illness in children. Future research should measure the true burden of *cdt*-positive *E. coli* diarrhoea among children.

## Abbreviations

aEPEC, atypical enteropathogenic Escherichia coli; CBC, complete blood count; CDT, cytolethal distending toxin; DAEC, diffusely adherent Escherichia coli ; DEP, diarrhoeagenic Escherichia coli pathotype; EAEC, enteroaggregative Escherichia coli; IBS, irritable bowel syndrome; IL, interleukin; ORS, oral rehydration therapy; PED, Paediatric Emergency Department ; tEPEC, typical enteropathogenic Escherichia coli.

## Introduction

Diarrhoeal illness is the main cause of mortality in Mexican children between 1 and 4 years of age [1]. As in other countries worldwide, diarrhoeagenic *Escherichia coli* pathotypes (DEPs) are the main causal agents of diarrhoea in children under 5 years of age, surpassing rotavirus, *Salmonella* and *Shigella* [2–5]. In a recent study, we found that most DEP and non-DEP *E. coli* strains isolated from children with diarrhoea in Mexico harboured other virulence factors, including toxins such as Cytolethal Distending Toxin (CDT) [4].

CDTs are heterotrimeric holotoxins, consisting of CdtA, CdtB and CdtC subunits, which have been identified in Gram-negative pathogenic bacteria [6]. CdtB corresponds to the active subunit, while both CdtA and CdtC form a heterodimeric subunit required for binding to target cells and intracellular delivery of the CdtB subunit [7]. CDTs are unique among bacterial toxins as they induce DNA double strand breaks in both proliferating and non-proliferating cells. CDT intoxication of epithelial cells leads to nuclear and cytoplasmic distension, formation of actin stress fibres and nuclear fragmentation, resulting in irreversible cell cycle arrest and death of target cells [7–9].

CDT-encoding genes have been identified in both diarrhoeagenic and uropathogenic *E. coli* strains, as well as in those that do not belong to any recognized pathotype [4, 10, 11]. There is scant information on the prevalence and clinical presentation of diarrhoeal disease caused by CDT-producing *E. coli* strains. We present five cases of young children with acute diarrhoea in whom a *cdt-*positive *E. coli* strain was the sole pathogen isolated from stools. One *cdt*-positive strain from each patient was tested in a cytotoxic assay to confirm CDT production and in a *cdt* type-specific PCR that identifies four *cdt* types (I, II, III, IV).

## Case report

The Hospital General O’Horan is a public hospital in Merida, Mexico, which receives patients from throughout the Yucatan Peninsula. The Paediatric Emergency Department (PED) has an oral rehydration unit with an active surveillance program for diarrhoeal pathogens that includes *Salmonella*, *Shigella*, *Vibrio* spp., *Campylobacter* and DEPs, as well as rotavirus and parasites. Diagnosis and management of acute diarrhoea in children is based on the Mexican Ministry of Health and WHO guidelines [12, 13]. All children at the oral rehydration unit are routinely assessed for vital signs, weight and height, as well as dehydration based on general condition, eyes, oral mucosa, thirst, respiratory rate, skin pinch, heart rate and capillary refilling time [13]. Based on the severity of the dehydration and the presence of complications, each child is assigned to rehydration treatment plan A, B or C and receives additional medications as deemed necessary by the attending physician. Assessment of nutritional status is determined by the WHO growth charts [14]. Weight measured after the correction of dehydration is used to calculate nutritional status.

The five children were identified during two different studies conducted from January 2007 to January 2009 and from January 2010 to July 2014. All of them sought medical care at the Hospital General O’Horan PED for acute diarrhoea. Both protocols were reviewed and approved by the Hospital Institutional Review Board, and written informed consent was obtained from all legal guardians to collect clinical samples and information for investigational studies. In all the children, stool cultures were exclusively positive for CDT-producing *E. coli* strains and negative for all other conventional pathogens (rotavirus, parasites or other bacteria). In two children, the *cdt-*positive strains isolated from stools were classified as typical (tEPEC) or atypical (aEPEC) enteropathogenic *E. coli*. The remaining three children harboured *cdt-*positive strains that did not belong to any diarrhoeagenic pathogroup. The salient features of the five cases and their *E. coli* strains are shown in Table 1.

**Table 1.** Clinical severity on hospital admission and strain characteristics of patients with CDT-producing positive *E. coli*

Case no.	Age (months)	Sex	No. of stools/24 h	No of vomiting episodes/24 h	Maximum temperature (°C)*	Hydration status	Total length of episode (days)	DEP	*bfp*A	*eae*A	*cdt*
1	14	M	20	6	40	Hypovolaemic shock	5	None	−	−	+
2	10	M	12	0	39.5	Moderate dehydration	4	None	−	−	+
3	38	F	7	8	38	No dehydration	5	tEPEC	+	+	+
4	3	M	9	1	38	Moderate dehydration	6	aEPEC	−	+	+
5	29	M	20	0	38.2	Moderate dehydration	5	None	−	−	−

F, Female; M, male.

*Temperature registered by mother at home or during the day of admission.

### Case 1

A previously healthy 14-month-old Mayan boy was admitted for hypovolaemic shock caused by 2 days of diarrhoea and vomiting. The child had presented 20 watery stools and 6 episodes of vomiting over the previous 24 h. His primary care physician had prescribed trimethoprim/sulphamethoxazole and acetaminophen, with no improvement. On examination, the child was somnolent with dry oral mucosa, sunken eyes, no tears on crying, very slow skin pinch and capillary refill of 6 s. He had not urinated in the previous 6 h. Vital signs were as follows: temperature, 40 °C; pulse, 160 beats min^−1^; respiration, 50 breaths min^−1^. His weight was 9 kg and length was 76 cm. His weight-for-age, length-for-age and weight-for length were between the 0 and −2 z scores and he was classified as adequately nourished. The complete blood count (CBC) reported: haemoglobin, 8.5 g dl^−1^; leukocytes, 6.8×10^3^ µl^−1^; neutrophils, 3.3×10^3^ µl^−1^, platelets, 27×10^3^ µl^−1^. Serum electrolytes were within normal ranges. His dehydration was classified as severe, with an estimated fluid deficit of 10–15 %. He received plan C treatment with two intravenous boluses of 0.9 % saline solution (20 ml kg^−1^ each in 20 min), followed by maintenance intravenous (IV) therapy at 180 mlkg^−1^day^−1^ for an additional 24 h. He was administered 225 mg ceftriaxone twice daily during his hospitalization and discharged on the third day when his stool pattern returned to normal and tolerated oral feedings. Antibiotic-susceptibility testing revealed that the *cdt*-positive *E. coli* isolate was resistant to gentamicin and amikacin. The total duration of the diarrhoeal episode was 5 days.

### Case 2

A previously healthy 10-month-old Mestizo boy was admitted with a 1 day history of 12 watery stools and fever. He had not received any previous medications. On examination, the child was irritable, had sunken eyes with no tears, dry oral mucosa and thirst. Abdominal distention and increased peristalsis was also observed. He had normal skin turgor and a capillary refill of 3 s. Vitals signs on admission were as follows: temperature, 38.5 °C; pulse, 140 beats min^−1^; and respiration, 46 breaths min^−1^. His weight was 8.6 kg and length was 72 cm. His weight-for-age and length-for-age were between the 0 and −2 z scores, and his weight-for-length between the 0 and −1 z scores. He was classified as adequately nourished. The CBC reported haemoglobin, 11.3 g dl^−1^; leukocytes, 13.8×10^3^ µl^−1^; neutrophils, 9.6×10^3^ µl^−1^; platelets, 356×10^3^ µl^−1^. His dehydration was classified as moderate, with an estimated fluid deficit of 5–10 %, and he was prescribed plan B with oral rehydration therapy (ORS) [(mEq l^−1^): Glucose 75; Sodium 75; Chloride 65; Potassium 20; Citrate 20. Osmolarity 245] at 100 mlkg^−1^ for 4 h. He presented three episodes of vomiting during the first 2 h and was switched to intravenous rehydration therapy with isotonic solution at 180 mlkg^−1^day^−1^ for 8 h; he also received one dose of 120 mg acetaminophen when his temperature reached 39.5 °C. He was discharged in good condition the next day and sent home with ORS and trimethoprim/sulphamethoxazole (64 mg/320 mg) for 5 days. The *E. coli* strain isolated from his stools was resistant to nalidixic acid and the β-lactam antibiotics ampicillin, ceftriaxone and ceftazidime, and susceptible to all other tested antibiotics, including trimethoprim/sulphamethoxazole. His stool pattern returned to normal after 4 days from onset.

### Case 3

A 38-month-old Mestizo girl with no concomitant illness was admitted for a 1 day history of fever, diarrhoea and vomiting. The mother referred that the child had presented seven watery stools and eight episodes of vomiting over the previous 24 h. The child had not received any medications. Physical examination on admission revealed a somnolent and irritable child with normal eyes and tears. The oral mucosa was moist and she was not thirsty. She had normal skin turgor and a capillary refill of <2 s. Vitals signs were as follows: temperature, 36 °C; pulse, 120 beats min^−1^; respiration, 26 breaths min^−1^. Her weight was 11.7 kg and her height 104 cm. Her weight-for-age was between the 0 and −2 z scores, and her height-for-age at the +2 z score. Her weight-for-height was below the −3 z score and was classified as severely wasted. As no dehydration was present, she was given plan A rehydration and was discharged in good condition after 6 h. She did not receive antibiotics or other medications. The *E. coli* strain was resistant to ampicillin, intermediately sensitive to nalidixic acid and susceptible to all other tested antibiotics. Her diarrhoeal episode lasted a total of 5 days.

### Case 4

A 3-month-old Mestizo boy was admitted for a 2 day history of diarrhoea and vomiting, with a mean of nine watery stools and one episode of vomiting over the previous 24 h. He had not received any medications. On examination, the child was irritable, with sunken eyes and no tears, with dry oral mucosa and intense thirst. The patient had decreased skin turgor and presented abdominal distension with decreased peristalsis. Capillary refill was 4 s. Vitals signs were as follows: temperature, 37 °C; pulse, 145 beats min^−1^; respiration, 30 breaths min^−1^. His weight was 3.4 kg and length 55 cm. His weight-for-age, length-for-age and weight-for-length were less than the −3 z score and he was classified as severely wasted. The CBC reported: haemoglobin, 11.5 g dl^−1^; leukocytes, 23.9×10^3^ µl^−1^; neutrophils, 16.8×10^3^ µl^−1^; platelets, 622×10^3^ µl^−1^. Electrolyte determination reported: Na, 130 mEq l^−1^; K, 2.5 mEq l^−1^. The patient was classified as having a fluid deficit of 5–10 % with moderate dehydration, and was assigned to receive plan B rehydration. He was administered two courses of ORS (WHO low-osmolarity ORS, same as Case 2), each at 100 mgkg^−1^ for 4 h and was discharged in good condition after 24 h. He was also given racecadotril (5 mg, three times daily) for 3 days and ampicillin (150 mg, four times daily) for 5 days. The antibiogram revealed that the *E. coli* strain was pansusceptible. The stool pattern was reported normal after 6 days from onset.

### Case 5

A 29-month-old Mestizo boy with a non-significant past medical history presented to the PED with fever and 5 days of diarrhoea with up to 20 stool movements per day. The mother had seen a primary care physician who had prescribed trimethoprim/sulphamethoxazole and nimesulide the previous day with little improvement. Physical examination revealed an irritable child with normal eyes and tears, dry oral mucosa, thirst, normal skin turgor and a capillary refill of <2 s. He also presented with abdominal distension and concentrated urine. Vitals signs were as follows: temperature, 38.2 °C; pulse, 144 beats min^−1^; respiration, 40 breaths min^−1^. His weight was 11 kg and his height 88 cm. His weight-for-age and height-for-age were between the 0 and −2 z scores; his weight-for-length was between the −1 and −2 z scores and he was classified as not malnourished. The CBC reported haemoglobin, 10.6 g dl^−1^; leukocytes, 11.8×10^3^ µl^−1^; neutrophils, 7.7×10^3^ µl^−1^; platelets, 248×10^3^ µl^−1^. The patient was classified as having a fluid deficit of 5–10 %, with moderate dehydration. He received one course of plan B rehydration (WHO low-osmolarity ORS, same as Cases 2 and 4) (100 mlkg^−1^ for 4 h) and was discharged in good condition with no medications. The *E. coli* isolate was pansusceptible. The total duration of his diarrhoeal episode was 5 days.

## Investigations

During the two study periods, stools samples from 1306 children with acute diarrhoea were collected in sterile containers with Cary Blair media inside them. Those children who presented dehydration on admission had blood samples taken for CBC and electrolyte analysis, as determined by the attending physician. Stools were inoculated into tetrathionate, Rappaport and alkaline peptone broths, and onto XLD (Xylose-Lysine-Desoxycholate), HE (Hektoen Enteric), BG (Brilliant Green), TCBS (Thiosulfate-Citrate-Bile salts-Sucrose), Cefex and MacConkey agars, and analysed by standard procedures [15]. Another set of stool samples was collected in empty sterile containers for microscopic ova and parasite examination, and detection of rotavirus. On arrival at the laboratory, a latex agglutination test was used for rapid detection of rotavirus (Pastorex Rotavirus; Bio-Rad); 1 g of stool was stored at −70 °C and later processed for rotavirus detection by ELISA (Premier Rotaclone; Meridian Bioscience). The five CDT-producing strains were tested for susceptibility to 12 different antibiotics (amikacin, ampicillin, ceftriaxone, ceftazidime, ciprofloxacin, chloramphenicol, gentamicin, imipenem, nalidixic acid, nitrofurantoin, trimethoprim/sulphamethoxazole and tetracycline) by disc diffusion according to Clinical and Laboratory Standards Institute guidelines [16].

Of the 1306 stool samples, 1060 (81.2 %) yielded *E. coli*-like colonies on MacConkey agar. Five such colonies were selected from each plate and biochemically confirmed as *E. coli*. All isolates were subjected to two previously described multiplex PCR assays [17, 18] that identify the DEP genes encoding heat-stable and heat-labile enterotoxins (*st*, *lt*) for ETEC, intimin (*eae*A) and bundle-forming pilus (*bfp*A) for tEPEC, intimin (*eae*A) for aEPEC, Shiga toxin 1 and 2 (*stx*1, *stx*2) and intimin (*eae*A) for Shiga toxin-producing *E. coli* (STEC), invasion-associated loci (*ial*) for enteroinvasive *E. coli* (EIEC), the AggR-master regulon (*agg*R), dispersin (*aap*) and the Aap translocator or anti-aggregative transporter (*aatA*) for enteroaggregative *E. coli* (EAEC), and Afa adhesin (*afa*C) for diffusely adherent *E. coli* (DAEC). An additional multiplex PCR assay was used to identify the presence of genes encoding EAST1 (*astA*), CDT (*cdt*B), Pet (*pet*) and subtilase (*sub*AB) toxins [4].

**Fig. 1.**
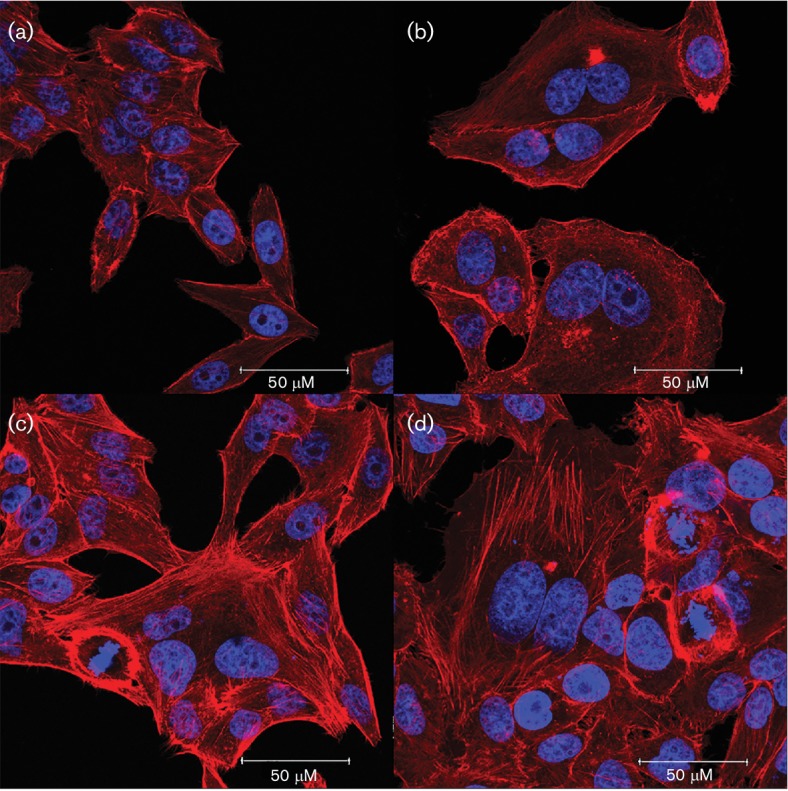
Cytotoxic effect of CDT produced by *E.coli* on HeLa cells. HeLa cells treated with *cdt*-positive sonicates of (b) O86 : H34 reference strain, (c) 12–149b patient isolate and (d) 12-265a patient isolate exhibited evident cell and nuclear distension, polynucleated cells, nuclear fragmentation and actin accumulation in structures resembling stress fibres, when compared with HeLa cells treated with a *cdt*-negative *E. coli* sonicate 11–85d (a). All photographs were taken at a magnification of 63x. Bars, 50 µm.

Thirteen patients (1 %) had *E. coli* strains harbouring *cdt* genes; only five of them were negative for all other tested pathogens. The remaining eight patients had *Shigella flexneri* (three), *Salmonella* Muenchen (one), enterotoxigenic *E. coli* (one), rotavirus (one), *Campylobacter coli* (one) and non-O1 *Vibrio cholerae* (one). One *cdt*-positive strain from each of the five patients was tested for CDT production by a modified HeLa cell cytotoxic assay using a *cdt*-positive reference strain O86 : H34 and a *cdt*-negative 11-85d isolate as positive and negative controls, respectively [19]. Briefly, an *E. coli* colony from LB agar was inoculated in 50 ml LB media and incubated in a shaking bath at 37 °C for 18 h. After centrifugation, the bacterial pellet was sonicated (five cycles of sonication for 35 s at 70 % amplitude, followed by no sonication for 35 s), filtered and the total protein concentration determined. One millilitre sterile bacterial sonicate was added to a well of 20 % confluent HeLa cells and incubated for 24 h. Cells were washed the next day and further incubated for 48 h. They were then fixed in paraformaldehyde, permeabilized and stained with rhodamine phalloidin and To-Pro-3 to label polymerized actin and DNA, respectively. All five sonicates exhibited the characteristic cytoskeletal, nuclear and morphological acute alterations of CDT intoxication (Fig. 1). These five *cdt*-positive *E. coli* strains were further characterized by a type-specific PCR assay that identifies *cdt*-*I*, *cdt*-*II*, *cdt*-*III* and *cdt*-*IV* types using a previously described method [20**﻿**]. Briefly, a colony was resuspended in 20 µl ultrapure water, which was boiled for 10 min, and then centrifuged at 14 000 r.p.m. for 5 min; 3 µl supernatant was used for the PCR. All PCRs contained a 0.2 mM mix of each deoxynucleoside triphosphate (Invitrogen), 1× *Taq* DNA polymerase buffer, 0.4 µM concentration each primer, 1. 5 mM MgCl_2_, and 1 U *Taq* polymerase. The amplification protocol was as follows: denaturation at 94 °C for 5 min, followed by 30 cycles of denaturation (94 °C, 1 min), annealing (55 °C, 1 min) and extension (72 °C, 1 min). A final extension was carried out at 72 °C for 10 min. PCR products were visualized in 1.5 % agarose gels stained with ethidium bromide.

All five strains produced cytoskeletal, nuclear and acute cellular morphological alterations characteristic of CDT intoxication. Isolates from cases 3 and 4 harboured genes for *cdt*-*I* type, case 1 for *cdt*-*III*, case 2 for *cdt*-I*V*, while the isolate from case 5 harboured concurrently genes for *cdt*-*I, cdt*-*II* and *cdt*-*III* types.

## Discussion

Our findings suggest that CDT-producing *E. coli* strains may cause abundant watery diarrhoea with fever and vomiting. In almost all the children (4/5) in our cases, the stool output and vomiting was high enough to cause dehydration, and two of the patients presented hypovolaemic shock and electrolyte imbalance. None presented bloody diarrhoea. We compared the clinical data of the CDT-producing *E. coli* diarrhoea patients versus the clinical database (2010–2014 study) for DAEC and EAEC diarrhoea patients, the two most prevalent pathotypes identified during this period (unpublished data). The comparison revealed that patients with CDT-producing *E. coli* had a mean stool output of 13.6 in 24 h, which was significantly higher (*P*<0.05) than that of patients infected with DAEC (7.9/24 h) and EAEC (6.9/24 h). Similarly, all our patients presented fever above 38 °C, compared to 44.8 % for EAEC and 35 % for DAEC patients. In contrast, no differences were observed for vomiting among the different patient groups. The mean numbers of vomiting episodes in 24 h were 5, 6.9 and 4.5 for CDT, DAEC and EAEC patients, respectively.

Only two other reports, from Bangladesh and India, describe the clinical symptoms in children with CDT-producing *E. coli* diarrhoea [21, 22]. The Bangladeshi study reported a 3.1 % prevalence of *cdt*-positive *E. coli*; none of their patients had blood in their stools [22]. All eight cases presented watery diarrhoea but of lesser severity than our children, as only 50 % had vomiting and there was only one case of dehydration or fever. The prevalence of CDT-producing *E. coli* in the Indian study (1.4 %), however, was similar to ours, although it is notable that more than half of the Indian children had bloody stools [21]. STEC CDT-producing strains have been identified [11]. Thus, it would be important to ascertain whether the isolates associated with bloody diarrhoea harboured other toxin genes such as Shiga toxins. Whether *E. coli* strains that produce CDT exclusively are associated with bloody diarrhoea warrants further investigation, as none of the *cdt*-positive patients in our study or the Bangladeshi study presented bloody diarrhoea.

All four *cdt*-types genes were identified among our strains; most of them (60 %) carried the *cdt-I* type that was also the predominant type in Japan [10]. The isolate from case 5 harboured genes for *cdt*-*I, cdt*-*II* and *cdt*-*III* types. The child in this case, as well as the child in case 1, presented the highest stool output per day (20 stools/24 h). Both children were adequately nourished, but compared to case 1, case 5 was double the age (29 vs 14 months). It is plausible that strains infecting older children may require production of several types of CDT toxin to produce severe disease. Two other *E. coli* isolates from case 5 were *cdt* typed and confirmed to harbour *cdt*-*I, cdt*-*II* and *cdt*-*III* genes. Future studies should determine whether all three CDT types are expressed by these strains.

Isolates from all children produced the characteristic cellular intoxication caused by CDT, which strongly suggests that this toxin may be responsible for the high stool output observed. CDT from *Helicobacter pullorum* has been shown to target vinculin present in intestinal epithelial cells, triggering an atypical delocalization of vinculin from focal adhesions coupled with decreased cellular adherence [23]. Vinculin is a cytoplasmic actin-binding protein that is a key component of both focal adhesions and adherens junctions [24]. Animal models of post-infectious *cdt*-positive *Campylobacter jejuni* have demonstrated that host antibodies to CdtB cross-react with vinculin in the host gut producing an irritable bowel syndrome (IBS)-like phenotype [25, 26]. Furthermore, studies in humans have confirmed that anti-CDT-B and anti-vinculin antibodies are elevated in patients with diarrhoea-predominant-IBS compared to non-IBS subjects with celiac disease or healthy controls. These antibodies have been proposed as biomarkers that may assist in distinguishing diarrhoea-predominant-IBS from inflammatory bowel disease in patients with chronic diarrhoea [25].

All our CDT-patients had fever, which is initiated when peripherally produced endogenous pyrogens, such as cytokines interleukin-1 beta (IL-1β), tumor necrosis factor alpha and IL-6, reach areas surrounding the hypothalamus [27]. These cytokines induce the synthesis of cyclooxygenase-2, which in turn leads to the production of prostaglandins that alter hypothalamic temperature control, leading to an increase in heat production, the conservation of heat and finally fever [27]. Purified CDT from *Aggregatibacter actinomycetemcomitans* stimulated the production of IL-6, but not IL-1β or tumor necrosis factor alpha cytokines, in gingival fibroblasts [28], and increased IL-1β, IL-12 and IL-10 production in macrophages [29]. Future research should elucidate whether CDT from *E. coli* actually induces the production of endogenous pyrogens in humans.

As this was a small observational study, it is difficult to determine whether antibiotics are useful for treating diarrhoea caused by CDT-producing *E. coli*. Two of the children were given trimethoprim/sulphamethoxazole before hospital admission with little improvement of their symptoms despite their strains being susceptible to this antibiotic. It should be noted, however, that both patients had received treatment for 1 day or less. It is noteworthy that for four of the five children their illness was perceived as severe enough to warrant antibiotics by their attending physician. Previous studies have shown that the use of antibiotics for bacterial diarrhoea, including toxin-producing *E. coli*, as enterotoxigenic *E. coli*, shortens the duration of the episode and prevents complications [30]. A larger study that considers variables such as the severity of illness, age, dosages of antibiotics and duration of treatment would be necessary to determine the efficacy of antibiotics for the management of CDT-producing *E. coli* diarrhoea.

Our study is to the best of our knowledge the first to report CBC data from children with CDT-producing *E. coli* diarrhoea. Although no specific pattern was apparent, two of the children presented leukocytosis with neutrophilia; notably, one of the children presented severe thrombocytopenia with 27×10^3^ platelets µl^−1^. Diarrhoea associated with severe thrombocytopenia has been reported in non-typhoidal *Salmonella* cases [31]. The specific mechanism for lowering the platelet count, either through a cytotoxic effect on bone marrow or through the destruction of circulating platelets, is not yet clear.

In conclusion, our study shows that CDT-producing *E. coli* strains may cause severe acute diarrhoea with high stool output, vomiting and fever. The patients in the five cases reported here harboured *cdt*-positive *E. coli* strains in their stools and were negative for other bacterial or other enteric pathogens. Sonicates from all five strains exhibited the hallmark features of CDT intoxication on HeLa cells. The true prevalence of this emerging pathogen should be the focus of future studies.
